# Clinical tolerance but no protective efficacy in a placebo-controlled trial of repeated controlled schistosome infection

**DOI:** 10.1172/JCI185422

**Published:** 2024-12-12

**Authors:** Jan Pieter R. Koopman, Emma L. Houlder, Jacqueline J. Janse, Olivia. A.C. Lamers, Geert V.T. Roozen, Jeroen C. Sijtsma, Miriam Casacuberta-Partal, Stan T. Hilt, M.Y. Eileen C. van der Stoep, Inge M. van Amerongen-Westra, Eric A.T. Brienen, Linda J. Wammes, Lisette van Lieshout, Govert J. van Dam, Paul L.A.M. Corstjens, Angela van Diepen, Maria Yazdanbakhsh, Cornelis H. Hokke, Meta Roestenberg

**Affiliations:** 1 Leiden University Center for Infectious Diseases,; 2 Department of Cell and Chemical Biology,; 3 Department of Clinical Pharmacy and Toxicology, and; 4 Center for Cell and Gene therapy, Leiden University Medical Center (LUMC), Leiden, Netherlands.

**Keywords:** Infectious disease, Parasitology

## Abstract

**BACKGROUND:**

Partial protective immunity to schistosomiasis develops over time, following repeated praziquantel (PZQ) treatment. Moreover, animals develop protective immunity after repeated immunization with irradiated cercariae. Here, we evaluated the development of natural immunity through consecutive exposure-treatment cycles with *Schistosoma mansoni* in healthy, *Schistosoma*-naive participants using single-sex, controlled human *S. mansoni* infection.

**METHODS:**

Twenty-four participants were randomized in a double-blinded (1:1) manner to either the reinfection group, which received 3 exposures (weeks 0, 9, and 18) to 20 male cercariae, or to the infection control group, which received 2 mock exposures with water (weeks 0 and 9) prior to cercariae exposure (week 18). Participants were treated with PZQ (or placebo) at weeks 8, 17, and 30. Attack rates (ARs) after the final exposure (weeks 19–30) using serum circulating anodic antigen (CAA) positivity were compared between groups. Adverse events (AEs) were collected for safety.

**RESULTS:**

Twenty-three participants completed the follow-up. No protective efficacy was observed, given an 82% (9 of 11) AR after the final exposure in the reinfection group and 92% (11 of 12) in the infection control group (protective efficacy 11%; 95% CI –24% to 35%; *P* = 0.5). Related AEs were higher after the first infection (45%) compared with the second (27%) and third (28%) infections. Severe acute schistosomiasis was observed after the first infections only (2 of 12 in the reinfection group and 2 of 12 in the infection control group).

**CONCLUSION:**

Repeated *Schistosoma* exposure and treatment cycles resulted in apparent clinical tolerance, with fewer symptoms reported following subsequent infections, but did not result in protection against reinfection.

**TRIAL REGISTRATION:**

ClinicalTrials.gov NCT05085470.

**FUNDING:**

European Research Council (ERC) Starting Grant (no. 101075876).

## Abstract 2

Repeated controlled* Schistosoma* exposure and treatment resulted in clinical tolerance, but did not result in protection to reinfection, which may take longer to develop.

## Introduction

Schistosomiasis, an infection with *Schistosoma* worms, causes considerable disease burden, with over 200 million people infected and another 800 million at risk of infection worldwide (1). While mass drug administration with praziquantel (PZQ) is widely used to reduce the infection burden, progress in disease control has stalled in certain areas, highlighting the need for additional control strategies such as vaccines. Vaccine research is encouraged by data suggesting that some level of immunity, but not full protection, i.e., sterile protection, to *Schistosoma* (re)infection is acquired after multiple infections. This includes epidemiological data from *Schistosoma*-endemic areas that show an age-dependent decrease in infection burden most likely due to partially decreased susceptibility to infection over time (2), as well as promising results of immunization studies with irradiated cercariae resulting in a 70%–80% worm burden reduction in rodent and nonhuman primate models (3). Despite such studies, our knowledge of what immune mechanisms result in (natural) immunity or, in other words, partial protection from infection, remains limited, and correlates of protection are not well defined and differ between studies (4–7). Previously, we established a controlled human infection model with schistosomes (CHI-S) and demonstrated that single-sex exposure to 20 male *Schistosoma mansoni* cercariae resulted in detectable infection in 82% (9 of 11) of individuals, based on serum circulating anodic antigen (CAA) detection, and resulted in few severe side effects. Moreover, CHI-S led to induction of high levels of schistosome-specific IgG1, which, in animal models, have been associated with protection against reinfection (7). We therefore used this CHI-S model to investigate (protective) immune responses to repeated exposure and treatment cycles, to measure the development of protective immunity in humans and investigate the safety of (repeated) exposure to male cercariae.

## Results

### Study population.

**Figure 1:**
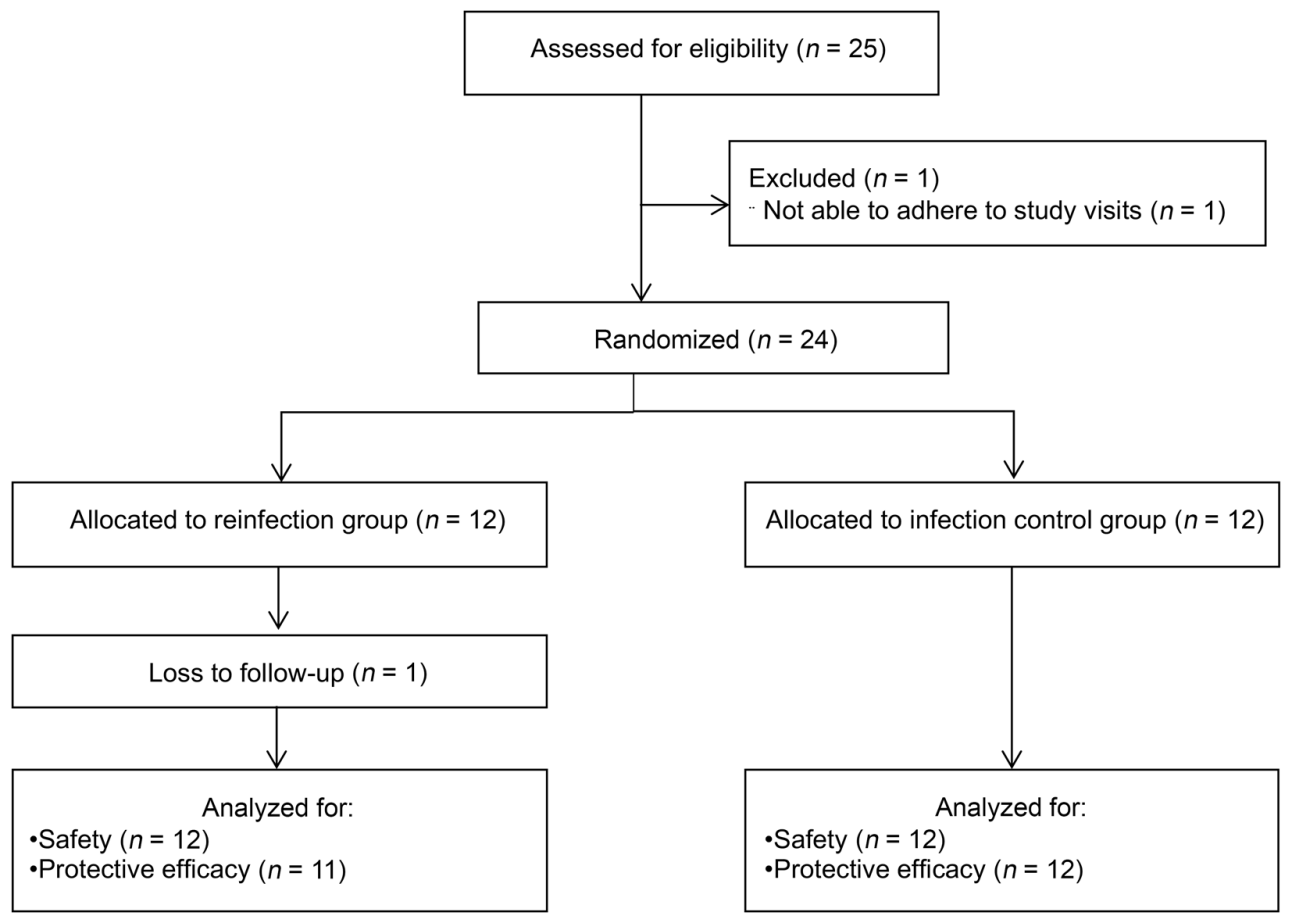
Consort flow for study participants.

In total, 25 individuals were screened for eligibility, 1 of whom was excluded because of the inability to attend all study visits (Figure 1). Twenty-four participants were randomly allocated to the reinfection group (*n* = 12) or the infection control group (*n* = 12). Participants in the reinfection group were exposed to 20 *S. mansoni* cercariae 3 times (weeks 0, 9, and 18), whereas the participants in the infection control group were only exposed once (week 18) and received 2 mock exposures (weeks 0 and 9). Treatment with 60 mg/kg PZQ (or placebo tablets for infection controls) was given 8 weeks after the first and second (mock) exposures and 12 weeks after the third exposure for all participants. One participant in the reinfection group was lost to follow-up shortly after the third exposure and was given PZQ treatment to clear the infection.

The median age of the participants was 23 years (range, 18–44 yr), 13 were female (54.2%), and the median BMI was 24.7 kg/m^2^ (range, 19.3–31.4) at baseline (Table 1). To monitor potential failed skin invasion, we performed microscopy on rinse water after each *Schistosoma* exposure and found very few remaining whole cercariae (range, 0–2) or heads (range, 0–3) (Supplemental Table 1; supplemental material available online with this article; https://doi.org/10.1172/JCI185422DS1).

### Safety.

Adverse event (AE) data were analyzed for all 24 participants. No serious AEs were reported. Over the course of the study, 246 related AEs were reported, of which 143 (58%), 66 (27%), and 37 (15%) were categorized as mild, moderate, and severe, respectively. Of these, 75% (*n* = 185) were associated with *Schistosoma* exposure, and 24% (*n* = 58) were common side effects of PZQ. The reinfection group reported 114 AEs related to *Schistosoma* exposure (Table 2), with the highest number reported after the first exposure (*n* = 51, 45%). After the second and third exposures, comparable numbers of AEs were reported (exposure 2: *n* = 31, 27%; exposure 3: *n* = 32, 28%). In the infection control group, most AEs related to *Schistosoma* exposure were reported after the third exposure (*n* = 45, 63%), although, notably, a considerable number of AEs were observed after the 2 initial mock exposures, suggesting a relatively high background incidence of these AEs (exposure 1: *n* = 8, 11%; exposure 2: *n* = 18, 25%).

The risk of PZQ-related AEs was similar after each treatment in the reinfection group (Supplemental Table 2), and very few AEs were reported after treatment with placebo in the infection control group (Supplemental Table 3).

**Figure 2:**
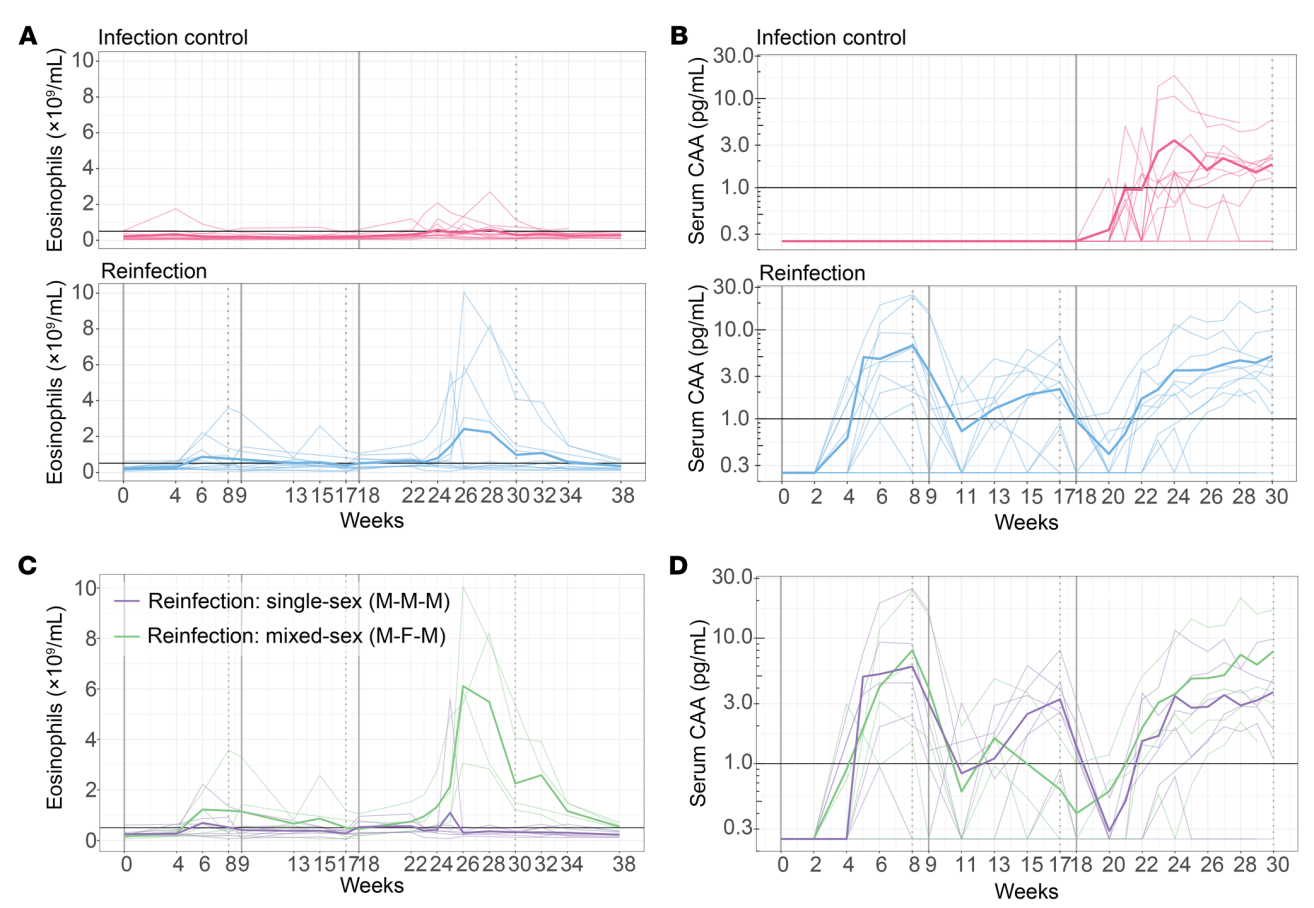
Eosinophil counts and CAA levels after (re)exposure to *S. mansoni* cercariae. Plots show the changes over time in eosinophils (**A**) and CAA (**B**) in infection control (pink, *n* = 12) and reinfection (blue, *n* = 12) participants. Eosinophils (**C**) and CAA (**D**) in the reinfection group were then plotted by stratification on the basis of whether single-sex (M-M-M) exposure (purple, *n* = 7) or accidental mixed-sex (M-F-M) exposure occurred. Individual participant data are plotted, with thicker lines showing the group means. The horizontal black line shows the cutoff for abnormal counts (≥0.5 × 10^9^/mL for eosinophils; ≥1.0 pg/mL for CAA). The solid, gray vertical line shows *S. mansoni* exposure weeks, while the gray, black vertical line shows when PZQ treatment was given.

Symptoms of *Schistosoma* exposure included local skin reactions as well as systemic responses (acute schistosomiasis [AS]) starting after 3 weeks. Systemic symptoms lasted a median of 1 day (IQR: <1–4 days). Clustering of symptoms was observed in some participants, suggestive of AS (Supplemental Figure 1). Severe AS (i.e., interfering with daily activities) was observed in 4 participants, and all instances occurred after the participant’s first (true) exposure — 2 in the infection control group and 2 in the reinfection group (Supplemental Table 4). Three were treated with 30 mg prednisolone for 5 days, with subsequent tapering of the dose (20 mg to 10 mg to 5 mg over the course of 1 week) to alleviate symptoms. Participants with severe AS after the first exposure in the reinfection group reported no (*n* = 1) or milder (*n* = 1, moderate) AEs after subsequent exposures. Eosinophil levels peaked in the reinfection group after the third exposure (Figure 2A). No clinically relevant changes in liver function tests were observed.

### Protective efficacy.

The attack rate (AR) based on CAA positivity after the third exposure in the reinfection group was 82% (9 of 11) and 92% (11 of 12) in the infection control group, corresponding to a protective efficacy of 11% with a wide 95% CI that included zero (–24% to 35%), indicating no protection (*P* = 0.5). The proportion of CAA-positive participants in the reinfection group after the first and second exposures was 64% (7 of 11) for both exposures. CAA levels over time did not decrease with subsequent exposures in the reinfection group (Figure 2B). There was no association between severe AS and CAA levels (Supplemental Figure 2). After treatment following the third exposure, 3 participants received additional PZQ treatment because of persistent CAA positivity 6 and/or 8 weeks after the exposure. Complete clearance of infection, i.e. negative CAA, was achieved in all participants and confirmed at a final visit 1 year after the start of the study.

### Accidental exposure to female cercariae and potential egg production.

Results of *Schistosoma* PCR on feces were all negative after the first and second exposures, however, after the third exposure, 1 participant had a positive result (CT ~32), indicating the presence of *Schistosoma* DNA and egg production, which was later confirmed by microscopy. The number of eggs found was low (6 eggs in 3 separate Ridley ×6 slides). All procedures for the production of challenge material were rechecked, and no irregularities in study processes were found. Upon molecular retesting of all stored cercariae used for infection, we discovered that 5 participants, during the second exposure, were accidentally exposed to 20 female, instead of male, cercariae due to sample mislabeling. We hypothesize that persistent single-sex females, which are more resistant to treatment with PZQ (8), after the second infection-treatment cycle in these individuals could have led to a patent egg-producing male-female worm pair after the third infection. The procedures were adapted, and a second molecular confirmation step was implemented to avoid such incidents in the future.

In post hoc analyses, participants with mixed-sex (male-female-male [M-F-M]) exposure had higher peak eosinophil counts after the third exposure compared with those with single-sex male (M-M-M) exposure (Figure 2C), but AEs and CAA positivity/kinetics did not seem to differ between the 2 groups (Figure 2D). Of the 3 participants requiring additional PZQ treatment, 2 were infection controls and 1 was a reinfection participant who was only exposed to male cercariae.

### Antibody, chemokine, and cytokine responses.

**Figure 3:**
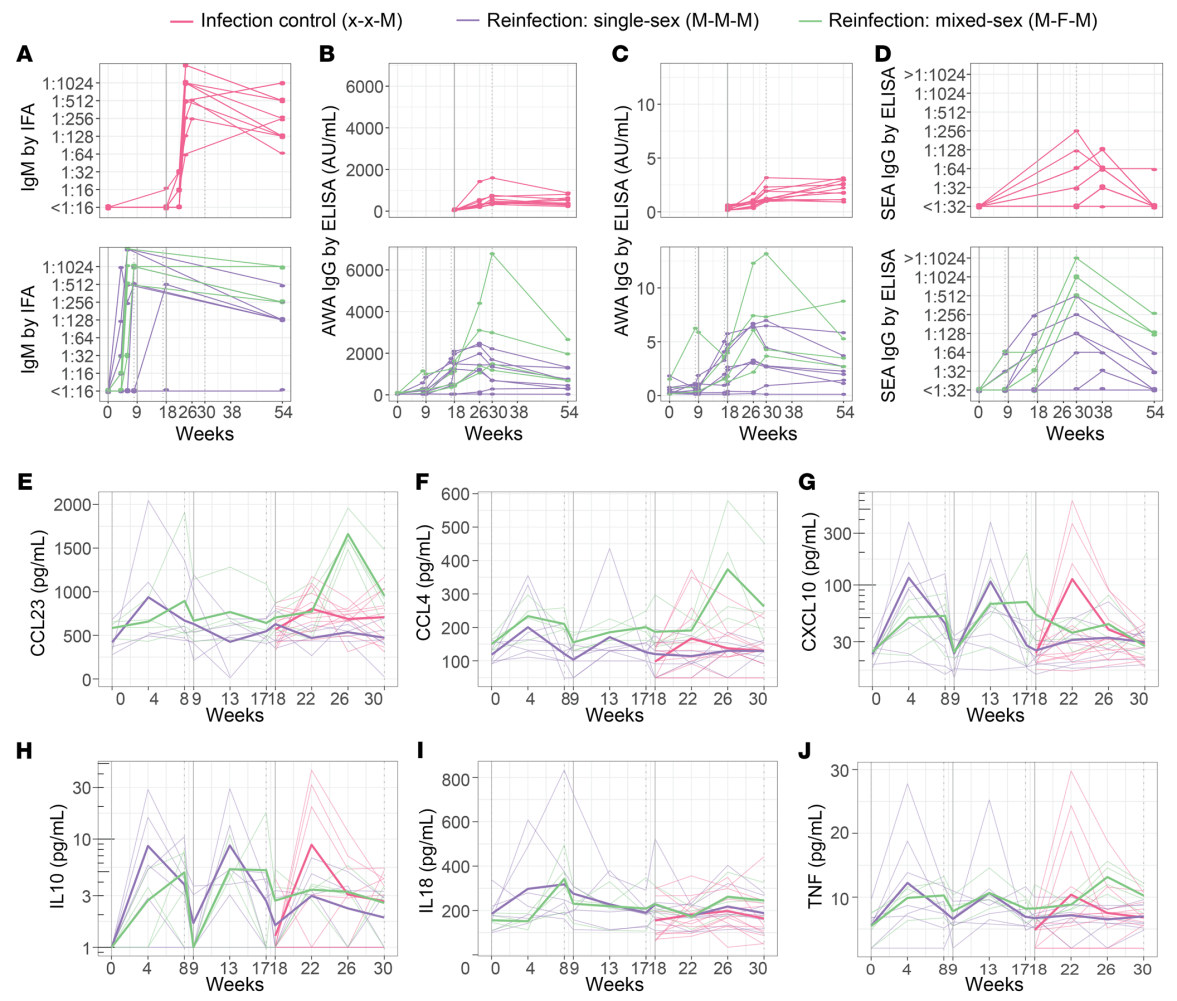
Antibody, chemokine, and cytokine responses after (re)exposure to *S. mansoni* cercariae. Plots show the individual changes in antibody levels in worm-specific IgM (**A**), AWA-specific IgG (**B**), AWA-specific IgG1 (**C**), and SEA IgG (**D**). For CCL23 (**E**), CCL4 (**F**), CXCL10 (**G**), IL-10 (**H**), IL18 (**I**), and TNF (**J**), individual participant data and group means (thicker lines) are plotted. Data were stratified for infection controls (pink, *n* = 12), reinfection single-sex (M-M-M) exposure (purple, *n* = 7), and reinfection accidental mixed-sex (M-F-M) exposure (green, *n* = 5). The solid gray vertical line shows *S. mansoni* exposure weeks (0, 9, and 18), and the dotted gray vertical line shows when PZQ treatment was given (weeks 8, 17, and 30).

M-F-M exposure appeared to influence the (egg-specific) antibody and cytokine response data, which are therefore presented separately. Within 8 weeks of the initial exposure to cercariae, 21 of 23 participants had seroconverted for worm-specific IgM (Figure 3A). One seroconverted later at week 18, while the other remained negative. IgG and IgG1 antibodies against adult worm antigen (AWA) increased after exposure in all but 1 participant. Peak levels in the reinfection group appeared to increase with subsequent exposures, suggesting a boost (Figure 3, B and C). Increases in IgG against soluble egg antigen (SEA) were observed in most participants, as previously also observed in male-only exposure, possibly due to antibody cross-reactivity between cercariae and eggs (9). However, those exposed to M-F-M cercariae had higher peak values than did those only exposed to M-M-M cercariae (Figure 3D).

Serum cytokines and chemokines showed similar kinetics after the first exposure in both the reinfection and infection controls (Figure 3, E–J), as none of these mean cytokine/chemokine levels differed between the groups 4 weeks after primary exposure. We observed some evidence that the levels of CCL4 were lower at week 22 (4 weeks after the third exposure) compared with week 4 (mean difference –70.3, 95% CI: –129.7; –11.3, *P* = 0.04). Although visually, CXCL10 and TNF levels also appeared lower after the third infection, we were unable to detect a statistically significant difference, potentially because of the small sample size. After the third exposure, in the reinfection group, CCL23 (*P* < 0.001), CCL4 (*P* = 0.05), and TNF (*P* < 0.001) levels were higher in the M-F-M–exposed individuals than in the M-M-M–exposed individuals. We observed no association between severe AS symptoms and circulating cytokines or chemokines (Supplemental Figure 3).

## Discussion

In this study, we demonstrate that repeated controlled exposure to *S. mansoni* cercariae did not lead to protection against reinfection but induced tolerance to clinical symptoms already after the first infection, with fewer AEs being reported after subsequent infections.

In line with previous CHI-S, local skin reactions (rash and itch) and systemic symptoms of AS were commonly observed, albeit of short duration, with severe AS reported in 4 of 24 individuals after the first exposure. This risk of severe AS after primary exposure is consistent between both the reinfection and infection control groups and across previous studies (8, 9). The risk of AS decreased with subsequent exposures, which may explain why AS is infrequently reported in endemic populations (10), in which exposure to *Schistosoma* antigens is thought to start at an early age, potentially even in utero (11), and occur further throughout life. In our earlier work, we have shown severe AS to be accompanied by a Th1-biased inflammatory response at week 4 (12), but we found no relationship between CAA and symptoms (8, 9), which was confirmed in the current study. Clinical tolerance is likely to be accompanied by regulatory responses, but further research will be needed to delineate the details of the underlying mechanisms.

Unlike in earlier CHI-S studies, here we included an infection control group that received mock infections with water. Both participants and investigators were masked to group allocation, resulting in a large number of AEs classified as potentially related to infection with *Schistosoma*, even after water exposure. This demonstrates that AS symptoms, e.g., abdominal symptoms or headache, were aspecific and have a high incidence in the general population, making AS diagnosis challenging. While individual symptoms were aspecific, our data indicate that, in particular, clustering of symptoms 4–5 weeks after challenge was highly suggestive of AS. By looking at the difference in risk of symptoms between those exposed to *Schistosoma* and water, we can now more reliably assess the safety of CHI-S. For future studies looking to establish the safety of a novel, controlled human infection model, the inclusion of an infection control group may be considered, especially if the expected symptoms are aspecific and common.

Contrary to our hypothesis, we did not observe any evidence for sterile protection based on serum CAA levels after 2 exposure and treatment cycles. Moreover, the CAA kinetics following the second and third exposure showed no sign of partial protection despite IgG1 boosting, as peak CAA values did not decrease with consecutive exposures. Our current understanding of resistance to reinfection in humans comes from epidemiological studies in endemic settings that suggest immunity can develop as a result of worm death and subsequent antigen release, as observed in occupationally exposed adults in endemic settings (13). Worm-specific IgG responses are associated with protection in animal immunization studies with irradiated cercariae (14) and with protection in endemic settings (15). Although some individual studies in endemic settings have suggested that higher levels of worm-specific IgE levels are protective, this could not be confirmed after meta-analysis (5). Apart from the infectious dose, which is much higher in animal studies (>1,000 cercariae) and in endemic settings, the apparent discrepancy between these studies and our findings could be explained by the quality and specificity of the IgG response. Perhaps the anti–worm IgG responses we observed were not against specific protective antigens on the worm or did not reach a high enough titer — two factors previously shown to be critical for protection (16, 17). Moreover, antibody functionality may also be shaped by the number of cumulative exposures, which, in endemic settings, are higher than in our study.

Several participants were accidentally exposed to M-F-M cercariae, for which we confirmed egg production in 1 participant, suggesting that (a) female worms were not fully cured with 60 mg/kg PZQ and (b) surviving female worms were able to pair with incoming male worms. Unlike female-only infection, where decreased susceptibility to PZQ is observed (8), the potential resulting mixed-sex and single-sex male infections responded well to PZQ, as only few participants (3 of 23) required a second dose of PZQ before being fully cured. Cure rates after initial treatment with 60 mg/kg PZQ were also higher compared with our previous male-only CHI-S study, in which 6 of 14 participants required an additional dose after being initially treated with 40 mg/kg PZQ (9).

CAA levels in those exposed to M-M-M and M-F-M cercariae did not differ, however, the composition of single versus paired worms could be determined. We noted several differences between potentially mixed-sex worm– versus single-sex worm–infected participants in the reinfection group. From our data, it seems that potential egg production was accompanied by higher eosinophil, CCL23, CCL4, and TNF levels, as well as higher IgG antibody titers against SEA. An increasing dominance of type 2 responses after egg production is well described (18, 19) and is evidenced here by the increase in eosinophils and CCL23 levels, a chemokine constitutively produced by eosinophils during type 2 inflammation (20, 21). Notably, the initial response to potential egg production is also characterized by the proinflammatory cytokines CCL4 and TNF, as previously reported in murine systems (22–24).

Although there are clear limitations of the CHI-S model in its comparability to natural infection, the fact that we did not find any protection suggests that the immune-regulatory potential of CHI-S may be much stronger than we originally envisioned. However, we note several methodological choices that may have affected the protection outcome. Compared with irradiated CHI-S, our strategy of PZQ treatment abrogated infection at a later time point, which may have allowed for more regulatory responses to develop. Additionally, the use of CHI-S of 1 sex only may also have limited the induction of immunity as well as the low number of CHI-S for immunization and the limited number of immunizations. To further investigate natural immunity, we are looking forward to CHI-S studies in preexposed individuals that will answer these questions. It is also good to note that, although we observed clinical tolerance, the study was not primarily powered to detect differences in AE incidence.

Altogether, this study shows the rapid induction of clinical tolerance to CHI-S and lack of protective immune responses despite induction of antibodies and boosting thereof. An in-depth study of the antigen specificity of these responses, the cellular immune environment, and egg-driven immune responses will not only boost our understanding of schistosome immune regulation, but also provide a starting point to narrow the selection of vaccine targets.

## Methods

### Sex as a biological variable.

The participant’s sex was self-reported and used for descriptive purposes and not for analyses. Cercarial sex (male or female) was determined using molecular techniques as described elsewhere (9, 25).

### Study design and participants.

This double-blind, placebo-controlled, randomized trial was performed at the LUMC (Netherlands) between November 2021 and September 2022.

Healthy participants, aged 18–45 years, without prior (suspected) exposure to CHI-S and without travel plans to *Schistosoma*-endemic regions during the study period were recruited from Leiden, Netherlands, and the surrounding area through advertising. Individuals were excluded if they had a history or evidence of any (preexisting) illness that could compromise their health during the study or influence the interpretation of the study results. Moreover, individuals with a known hypersensitivity or contraindications to the rescue medication (PZQ, artesunate, or lumefantrine) were also excluded.

### Randomization and masking.

Participants were randomized to the reinfection or infection control group at a 1:1 ratio using a randomization list. Randomization was performed by a researcher independent of the study team. The participants and study team were blinded to group allocation.

### Study procedures.

The reinfection group was exposed to 20 *S. mansoni* cercariae 3 times (weeks 0, 9, and 18), while the infection control group was only exposed once (week 18) and received 2 mock exposures (weeks 0 and 9). Single-sex cercariae were produced as described previously (9, 25). In brief, snails were infected with a single *S. mansoni* miracidium, resulting in a monosexual infection. After 5 weeks, infected snails started shedding cercariae that were either male or female. The sex of these cercariae was determined using molecular techniques. These cercariae were then applied to the participant’s forearm in 0.5 mL mineral water for 30 minutes to mimic the natural route of infection. Next, the rinse water was checked for remaining cercarial heads and/or tails by microscopy by a laboratory technician, independent from the clinical team. After each (mock) exposure, participants were followed up frequently for AEs and sample collection to determine infection status. Treatment with 60 mg/kg PZQ (or placebo tablets for infection controls) was given 8 weeks after the first and second (mock) exposure. All participants were treated with 60 mg/kg PZQ 12 weeks after the third exposure and monitored afterwards for treatment success. Treatment was repeated in persistent infections (CAA ≥1.0 pg/mL).

### Outcomes.

The primary outcomes were (a) the protective efficacy of repeated exposure to male *S. mansoni*, measured as the difference in frequency of serum CAA positivity (≥1.0 pg/mL) between the reinfection and infection control groups after the third exposure; and (b) the frequency and severity of AEs after (repeated) exposure to male *S. mansoni* cercariae.

To determine infection status, worm-derived CAA was measured in 0.5 mL serum using the upconverting reporter particle lateral flow assay (UCP-LF CAA) as described previously (9, 26). Participants were considered infected if they had at least 1 CAA value of 1.0 pg/mL or higher before PZQ treatment. CAA values below the lower limit of detection of the assay (<0.5 pg/mL) were set to 0.25 pg/mL. CAA was measured retrospectively on serum samples after treatment of the third exposure in order to prevent deblinding.

To determine the safety of (repeated) exposures, AEs were collected and blood tests were performed. AEs were graded for severity and relatedness. Severity was assigned in 3 levels: symptoms that did not interfere with daily activities (mild); symptoms that interfered or limited daily activities (moderate); and symptoms that resulted in absenteeism or required bed rest (severe). Relatedness of AEs was assessed on the basis of clinical judgment, taking into account chronology, the timing of the event, and alternative diagnoses. In addition, we ascribed these related AEs to either schistosome exposure, drug treatment, or the study procedure (e.g., blood draws). We differentiated local (immediate) exposure site symptoms (rash, itch) and symptoms of AS. AS symptoms included (a combination of) fever, urticaria and angioedema, night sweats, myalgia, arthralgia, dry cough, diarrhea, abdominal pain, and headache, occurring between 2 and 12 weeks of exposure without any other clear cause. Safety blood tests included eosinophil counts and liver enzyme assessment. Fecal samples were assessed for *Schistosoma* DNA by PCR after each exposure, before treatment (27). In addition, we measured worm-specific IgM (immunofluorescence assay [IFA]) and SEA-specific IgG (ELISA) antibodies in serum using our in-house diagnostic assays (9, 28). AWA-specific IgG and IgG1 levels were measured using ELISA. Ninety-six-well half-area, high-binding microplates (Corning) were coated overnight at 4°C with 25 μg/mL AWA, prepared as described previously (29) in 0.1 M sodium carbonate buffer (pH 9.6). Plates were washed 3 times with washing buffer (0.05% Tween 20 in PBS) and blocked with 5% skimmed milk in PBS for 2 hours at room temperature. Plasma samples were serially diluted 2.5 times in 0.5% skimmed milk (1:100 to 1:12,500). After 3 washes, diluted plasma samples were added to the plate and incubated at room temperature for 2 hours. After 5 washes, plates were incubated with goat anti–human IgG (1:5,000) or mouse anti–human IgG1 (1:300, Thermo Fisher Scientific) conjugated with HRP (in 0.5% skim milk, 0·05% EDTA in PBS) for 1 hour at room temperature. After 6 washes, 3,3′,5,5′-tetramethylbenzidine (TMB) substrate was added. The reaction was stopped with 10% sulfuric acid after color development. Plates were read at 450 nm, with 570 nm used as a reference measurement and subtracted. Measurements were normalized to a standard curve consisting of polyclonal IgG (Merck) and expressed as AU/mL.

We used a custom Luminex kit to measure CCL4, CXCL10, IL-5, IL-13, TNF, CCL23, IFN-γ, IL-10, and IL-18 (Bio-Techne). Cytokines were included in the analysis if over 40% of samples were above the lower limit of detection. Three cytokines were excluded from the analysis — IL-5, IL-13, and IFN-γ, as they were detectable in less than 5% of all samples.

### Statistics.

Based on the previously determined AR of 82% after exposure to 20 male cercariae (9), we calculated that 11 participants would be required in each group to detect a 70% relative reduction in CAA positivity with 80% power and (2-sided) α = 0.05 significance level. The effect size was based on earlier studies in nonhuman primates which showed that immunization with irradiated cercariae led to a 70%–80% reduction in worm burden (30, 31). To account for loss to follow-up, we aimed to include 24 participants, 12 in each group. The AE data were analyzed in the intention-to-treat group (*n* = 24), and protective efficacy was analyzed in the per-protocol group (*n* = 23) consisting of participants who completed follow-up until week 30 and calculated similarly to vaccine efficacy estimates (1-RR or 1 – AR_reinfection_/AR_infection controls_) with corresponding 95% CIs. Data analysis and visualization were performed using R (version 4.3) and R Studio (version 2023.06.1). Cytokine levels between infection controls and reinfected participants were compared using unpaired, 2-tailed *t* tests, whereas differences in cytokine levels 4 weeks after the first and third exposure in the reinfection group were assessed using linear mixed models, with participant as a random effect and time in weeks as a fixed effect (as a factor) using packages lme4 (version 1.1–35) and lmerTest (version 3.1–3).

### Study approval.

Ethics approval was obtained from the local ethics review committee (Medisch-Ethische Toetsingscommissie Leiden Den Haag Delft [METC LDD], P21.070) and registered prospectively at ClinicalTrials.gov (NCT05085470). The study was conducted in accordance with the International Council for Harmonisation of Technical Requirements for Pharmaceuticals for Human Use (ICH) Guidelines for Good Clinical Practice and Declaration of Helsinki principles. Prior to any study procedure, informed consent was obtained from all participants.

### Data availability.

Individual data underlying the figures presented in this manuscript are available in the Supporting Data Values file. After publication, all data will undergo FAIRification (https://www.go-fair.org/fair-principles/fairification-process/) and will be made available, anonymized, through a LUMC-based FAIR data point. Contact the corresponding author for inquiries.

## Author contributions

MR acquired funding. JPRK and MR prepared the research protocol. JPRK, ELH, MR, CHH, and MY were involved in the study design. ELH, JCS, MCP, MYECVDS, IMVAW, and AVD were involved in the production and release of cercariae. JPRK, ELH, JJJ, OACL, GVTR, and STH generated the data. EATB, LJW, LVL, GJVD, and PLAMC were involved in the infection endpoint measurements and interpretation. JPRK and JJJ were involved in data curation and project administration. JPRK and ELH performed the data analyses and prepared the first draft of the manuscript. All authors have read and approved the final version of the manuscript.

## Supplementary Material

Supplemental data

ICMJE disclosure forms

Supporting data values

## Figures and Tables

**Table 1:**
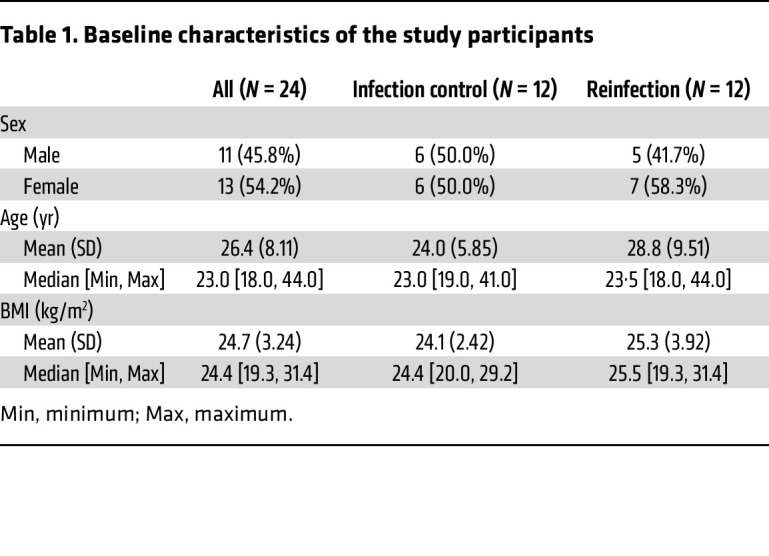
Baseline characteristics of the study participants

**Table 2:**
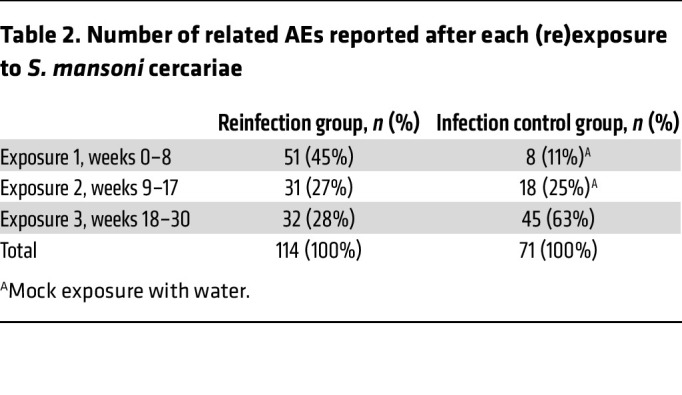
Number of related AEs reported after each (re)exposure to *S. mansoni* cercariae

## Version 1 12/12/2024

In-Press Preview

## Version 2 02/17/2025

Electronic publication
