# Supplementary material for: Clinical tolerance but no protective efficacy in a placebo-controlled trial of repeated controlled schistosome infection
Source: J Clin Invest. 2024 Dec 12;135(4):e185422. doi: 10.1172/JCI185422 (PMC11827845; doi:10.1172/JCI185422)
Supplement: Supplemental data [file jci-135-185422-s120.pdf]

**Supplementary material**

## Contents

Supplementary Table S1. Microscopy counts of cercariae in rinse water after exposure. 2

Supplementary Table S2. Risk of PZQ-related symptoms after treatment. 3

Supplementary Table S3. Number of related AEs reported after each PZQ treatment. 4

Supplementary Table S4. Risk of *Schistosoma*-related symptoms after (re)infection. 5

Supplementary Fig. S1. Severity and type of adverse events after Schistosoma (re)exposure stratified by group and exposure. 7

Supplementary Fig. S2 CAA levels and eosinophil counts after (re)exposure to Sm cercariae, stratified by severe acute schistosomiasis symptoms 8

Supplemental Fig. S3: Antibody, chemokine, and cytokine responses after (re)exposure to *Sm* cercariae, stratified by severe acute schistosomiasis symptoms 9

## Supplementary Table S1. Microscopy counts of cercariae in rinse water after exposure.

| | | Reinfection group (n=12), median (range) | Infection control group (n=12) , median (range) |
|---|---|---|---|
| **Exposure 1 Week 0** | Heads<br>Tails<br>Whole cercariae | 0.5 (0-1)<br>8.5 (2-15)<br>0 (0-2) | 0 (0-0)*<br>0 (0-0)*<br>0 (0-0)* |
| **Exposure 2 Week 9** | Heads<br>Tails<br>Whole cercariae | 0 (0-2)<br>5 (1-8)<br>0.5 (0-1) | 0 (0-0)*<br>0 (0-0)*<br>0 (0-0)* |
| **Exposure 3 Week 18** | Heads<br>Tails<br>Whole cercariae | 0 (0-3)<br>7 (5-14)<br>0 (0-2) | 0 (0-2)<br>4 (1-8)<br>0.5 (0-2) |

* Mock exposures with water.

## Supplementary Table S2. Risk of PZQ-related symptoms after treatment.

| | Reinfection (n=12) | | | Infection control (n=12) | | |
|---|---|---|---|---|---|---|
| | **Treatment 1** | **Treatment 2** | **Treatment 3** | **Treatment 1*** | **Treatment 2*** | **Treatment 3** |
| Any PZQ symptom# | | | | | | |
| Mild | 2 (17%) | 2 (17%) | 3 (25%) | 1 (8%) | 0 | 3 (25%) |
| Moderate | 2 (17%) | 4 (33%) | 2 (17%) | 0 | 0 | 1 (8%) |
| Severe | 4 (33%) | 0 | 1 (8%) | 0 | 0 | 1 (8%) |
| Abdominal Pain | | | | | | |
| Mild | 0 | 0 | 0 | 0 | 0 | 0 |
| Moderate | 0 | 0 | 0 | 0 | 0 | 1 (8%) |
| Severe | 0 | 0 | 0 | 0 | 0 | 0 |
| Nausea | | | | | | |
| Mild | 2 (17%) | 1 (8%) | 2 (17%) | 1(8%) | 0 | 3 (25%) |
| Moderate | 1 (8%) | 2 (17%) | 0 | 0 | 0 | 0 |
| Severe | 2 (17%) | 0 | 0 | 0 | 0 | 1(8%) |
| Heartburn | | | | | | |
| Mild | 0 | 0 | 0 | 0 | 0 | 0 |
| Moderate | 0 | 0 | 0 | 0 | 0 | 0 |
| Severe | 0 | 0 | 0 | 0 | 0 | 1(8%) |
| Dizziness | | | | | | |
| Mild | 2 | 2 (17%) | 3 (25%) | 0 | 0 | 1(8%) |
| Moderate | 1(8%) | 2 (17%) | 2 (17%) | 0 | 0 | 1(8%) |
| Severe | 4 | 0 | 0 | 0 | 0 | 0 |
| Disturbance of smell or taste | | | | | | |
| Mild | 0 | 0 | 1(8%) | 0 | 0 | 0 |
| Moderate | 0 | 0 | 0 | 0 | 0 | 0 |
| Severe | 0 | 0 | 0 | 0 | 0 | 0 |
| Fever | | | | | | |
| Mild | 0 | 0 | 0 | 0 | 0 | 0 |
| Moderate | 0 | 0 | 0 | 0 | 0 | 0 |
| Severe | 0 | 0 | 1(8%) | 0 | 0 | 0 |
| Headache | | | | | | |
| Mild | 0 | 0 | 1(8%) | 0 | 0 | 0 |
| Moderate | 0 | 0 | 0 | 0 | 0 | 0 |
| Severe | 0 | 0 | 0 | 0 | 0 | 0 |
| Malaise/fatigue | | | | | | |
| Mild | 0 | 0 | 0 | 0 | 0 | 0 |
| Moderate | 0 | 4 (33%) | 0 | 0 | 0 | 0 |
| Severe | 3 (25%) | 0 | 1(8%) | 0 | 0 | 1(8%) |
| Loss of appetite | | | | | | |
| Mild | 0 | 0 | 0 | 0 | 0 | 0 |
| Moderate | 0 | 1(8%) | 0 | 0 | 0 | 0 |
| Severe | 1(8%) | 0 | 0 | 0 | 0 | 0 |

* Treatment with placebo
# Only maximum severity counted
PZQ: praziquantel

## Supplementary Table S3. Number of related AEs reported after each PZQ treatment.

| | Reinfection group, n (%) | Infection control group, n (%) |
|---|---|---|
| **Treatment 1, week 8** | 17 (42%) | 3 (18%)* |
| **Treatment 2, week 17** | 13 (31%) | 0* |
| **Treatment 3, week 30** | 14 (27%) | 14 (82%) |
| **Total** | 41 (100%) | 17 (100%) |

* treatment with placebo
PZQ = praziquantel

## Supplementary Table S4. Risk of *Schistosoma*-related symptoms after (re)infection.

| | Reinfection (n=12) | | | Infection control (n=12) | | |
|---|---|---|---|---|---|---|
| | Exposure 1 | Exposure 2 | Exposure 3 | Exposure 1* | Exposure 2* | Exposure 3 |
| Any local symptom# | | | | | | |
| Mild | 10 (83%) | 11 (92%) | 8 (67%) | 0 | 4 (33%) | 7 (58%) |
| Moderate | 0 | 0 | 0 | 0 | 1 (8%) | 0 |
| Severe | 0 | 0 | 1 (8%) | 0 | 0 | 0 |
| Rash | | | | | | |
| Mild | 9 (75%) | 11 (92%) | 8 (67%) | 0 | 3 (25%) | 7 (58%) |
| Moderate | 0 | 0 | 0 | 0 | 0 | 0 |
| Severe | 0 | 0 | 0 | 0 | 0 | 0 |
| Itch | | | | | | |
| Mild | 9 (75%) | 2 (17%) | 7 (58%) | 0 | 3 (25%) | 5 (42%) |
| Moderate | 0 | 0 | 0 | 0 | 1 (8%) | 0 |
| Severe | 0 | 0 | 1 (8%) | 0 | 0 | 0 |
| Any systemic symptom# | | | | | | |
| Mild | 3 (25%) | 2 (17%) | 1 (8%) | 0 | 1 (8%) | 2 (17%) |
| Moderate | 2 (17%) | 3 (25%) | 1 (8%) | 1 (8%) | 2 (17%) | 2 (17%) |
| Severe | 2 (17%) | 1 (8%) | 2 (17%) | 1 (8%) | 1 (8%) | 2 (17%) |
| Acute schistosomiasis | | | | | | |
| Mild | 0 | 0 | 0 | 0 | 0 | 1 (8%) |
| Moderate | 1 (8%) | 2 (17%) | 1 (8%) | 0 | 0 | 1 (8%) |
| Severe | 2 (17%) | 0 | 0 | 0 | 0 | 2 (17%) |
| Fever | | | | | | |
| Mild | 0 | 0 | 0 | 0 | 1 (8%) | 1 (8%) |
| Moderate | 0 | 0 | 0 | 0 | 1 (8%) | 0 |
| Severe | 1 (8%) | 0 | 0 | 0 | 0 | 2 (17%) |
| Chills | | | | | | |
| Mild | 0 | 0 | 0 | 0 | 0 | 1 (8%) |
| Moderate | 0 | 0 | 0 | 0 | 0 | 0 |
| Severe | 0 | 0 | 1 (8%) | 0 | 0 | 0 |
| Night sweats | | | | | | |
| Mild | 0 | 0 | 0 | 0 | 0 | 0 |
| Moderate | 0 | 0 | 0 | 0 | 0 | 0 |
| Severe | 1 (8%) | 0 | 0 | 0 | 0 | 0 |
| Headache | | | | | | |
| Mild | 0 | 0 | 0 | 0 | 0 | 1 (8%) |
| Moderate | 0 | 0 | 1 (8%) | 1 (8%) | 0 | 1 (8%) |
| Severe | 1 (8%) | 1 (8%) | 0 | 0 | 0 | 2 (17%) |
| Malaise/fatigue | | | | | | |
| Mild | 2 (17%) | 3 (25%) | 0 | 1 (8%) | 0 | 1 (8%) |
| Moderate | 2 (17%) | 1 (8%) | 2 (17%) | 0 | 1 (8%) | 2 (17%) |
| Severe | 1 (8%) | 1 (8%) | 0 | 0 | 0 | 1 (8%) |
| Myalgia | | | | | | |
| Mild | 0 | 0 | 0 | 0 | 0 | 1 (8%) |
| Moderate | 2 (17%) | 1 (8%) | 0 | 0 | 0 | 0 |
| Severe | 1 (8%) | 0 | 0 | 0 | 0 | 1 (8%) |
| Urticaria | | | | | | |
| Mild | 0 | 0 | 1 (8%) | 0 | 0 | 1 (8%) |
| Moderate | 0 | 0 | 0 | 0 | 0 | 0 |
| Severe | 0 | 0 | 0 | 0 | 0 | 0 |
| Facial oedema | | | | | | |
| Mild | 0 | 0 | 0 | 0 | 0 | 0 |
| Moderate | 0 | 0 | 0 | 0 | 0 | 1 (8%) |
| Severe | 0 | 0 | 0 | 0 | 0 | 0 |
| Lymphadenitis | | | | | | |
| Mild | 0 | 0 | 0 | 0 | 0 | 1 (8%) |
| Moderate | 0 | 0 | 0 | 0 | 0 | 0 |
| Severe | 0 | 0 | 0 | 0 | 0 | 0 |
| Cough | | | | | | |
| Mild | 0 | 0 | 1 (8%) | 0 | 1 (8%) | 1 (8%) |
| Moderate | 0 | 0 | 0 | 0 | 1 (8%) | 0 |
| Severe | 0 | 0 | 0 | 0 | 0 | 0 |
| Sore throat | | | | | | |
| Mild | 0 | 0 | 0 | 0 | 2 (17%) | 1 (8%) |
| Moderate | 1 (8%) | 1 (8%) | 1 (8%) | 0 | 0 | 0 |
| Severe | 0 | 0 | 0 | 0 | 0 | 0 |

| | Reinfection (n=12) | | | Infection control (n=12) | | |
|---|---|---|---|---|---|---|
| | **Exposure 1** | **Exposure 2** | **Exposure 3** | **Exposure 1*** | **Exposure 2*** | **Exposure 3** |
| Loss of appetite | | | | | | |
| Mild | 2 (17%) | 1 (8%) | 0 | 0 | 0 | 0 |
| Moderate | 0 | 0 | 0 | 0 | 0 | 1 (8%) |
| Severe | 0 | 0 | 1 (8%) | 0 | 0 | 0 |
| Nausea | | | | | | |
| Mild | 1 (8%) | 1 (8%) | 0 | 0 | 0 | 0 |
| Moderate | 1 (8%) | 1 (8%) | 0 | 0 | 0 | 0 |
| Severe | 0 | 0 | 0 | 1 (8%) | 0 | 0 |
| Abdominal pain | | | | | | |
| Mild | 0 | 0 | 0 | 0 | 0 | 0 |
| Moderate | 1 (8%) | 2 (17%) | 0 | 1 (8%) | 0 | 1 (8%) |
| Severe | 0 | 0 | 1 (8%) | 0 | 1 (8%) | 0 |
| Diarrhoea or changed bowel habit | | | | | | |
| Mild | 3 (25%) | 1 (8%) | 0 | 1 (8%) | 0 | 1 (8%) |
| Moderate | 0 | 1 (8%) | 0 | 0 | 1 (8%) | 0 |
| Severe | 0 | 0 | 0 | 1 (8%) | 1 (8%) | 0 |
| Other | | | | | | |
| Mild | 0 | 1 (8%) | 1 (8%) | 0 | 0 | 1 (8%) |
| Moderate | 1 (8%) | 0 | 1 (8%) | 0 | 0 | 0 |
| Severe | 0 | 0 | 0 | 0 | 0 | 0 |

* Mock exposure with water
# Only maximum severity counted

**Supplementary Fig. S1. Severity and type of adverse events after Schistosoma (re)exposure stratified by group and exposure.**

A. Reinfection group

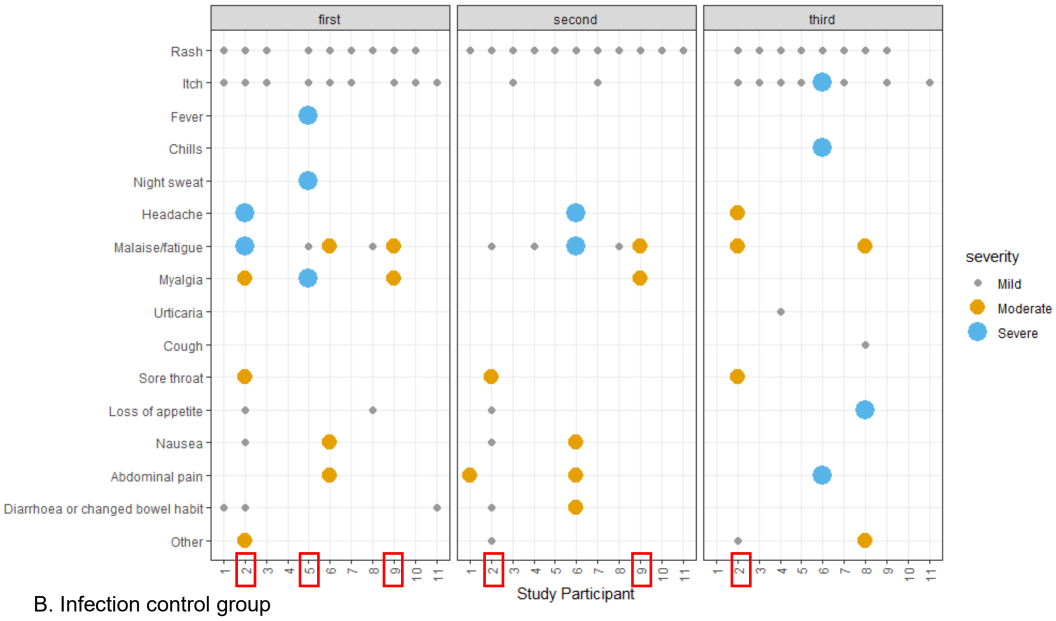


B. Infection control group

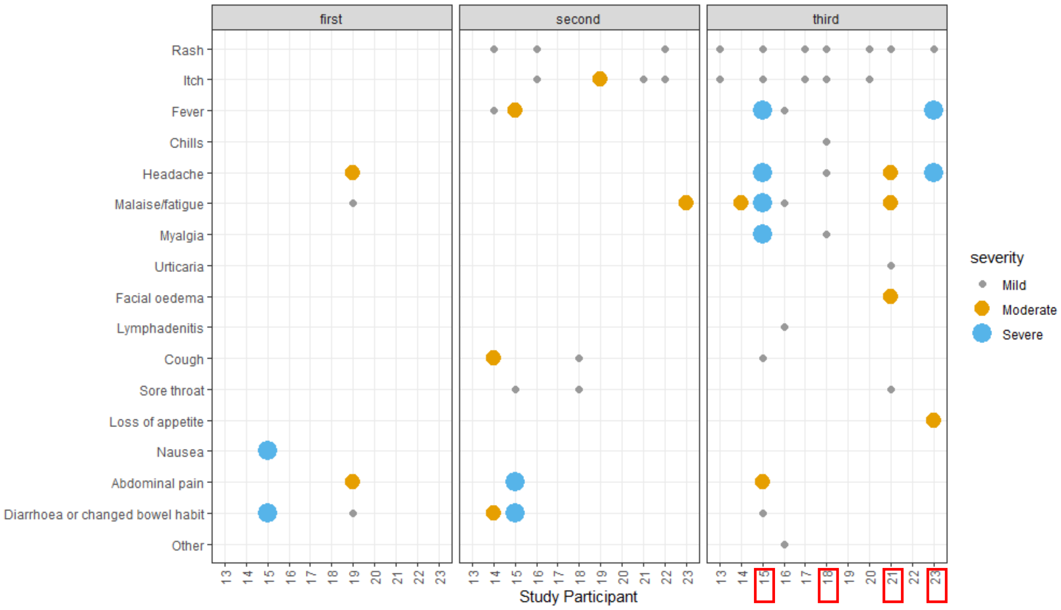


Graphs shows the incidence of local and systemic Schistosoma-related adverse events. It shows clustering of symptoms in particular participants, suggestive of acute schistosomiasis (in red boxed). Only the maximum severity for each adverse event after each exposure is plotted. Note that participants 12 and 24 did not report related adverse events and are therefore not shown in the graph.

**Supplementary Fig. S2 CAA levels and eosinophil counts after (re)exposure to Sm cercariae, stratified by severe acute schistosomiasis symptoms**

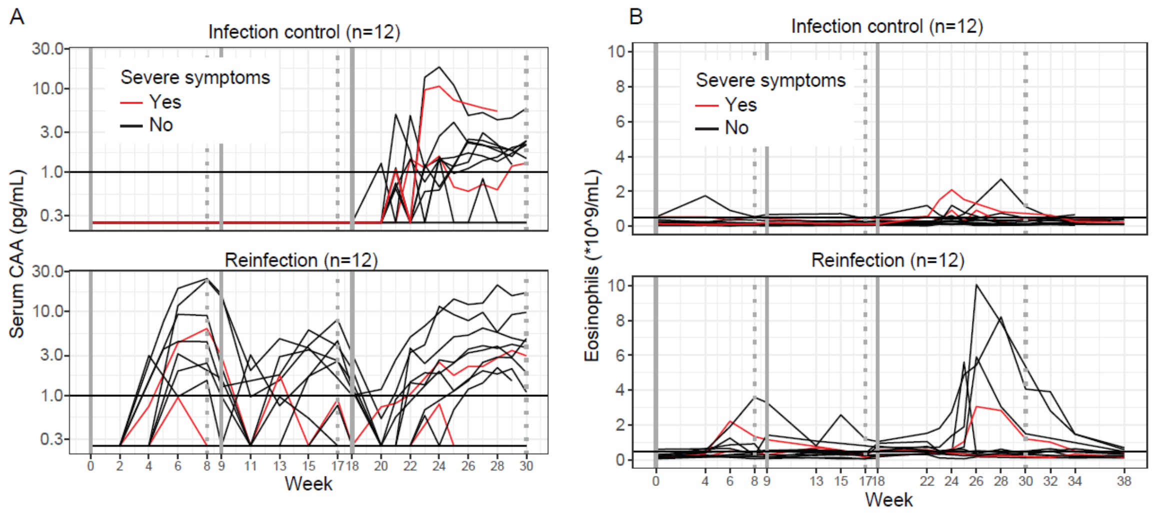


Plots show the changes over time in CAA (A) and eosinophils (B) in infection control (n=12) and reinfection (n=12) participants stratified by severe acute schistosomiasis symptoms (yes, n=4). Individual participant data is plotted. The horizontal black line shows the cut-off for abnormal counts (≥0.5 *10^9/mL for eosinophils; ≥1.0 pg/mL for CAA). The solid, grey vertical line shows *Sm* exposure weeks, while the grey, black vertical line shows when PZQ treatment was given.

**Supplemental Fig. S3: Antibody, chemokine, and cytokine responses after (re)exposure to *Sm* cercariae, stratified by severe acute schistosomiasis symptoms**

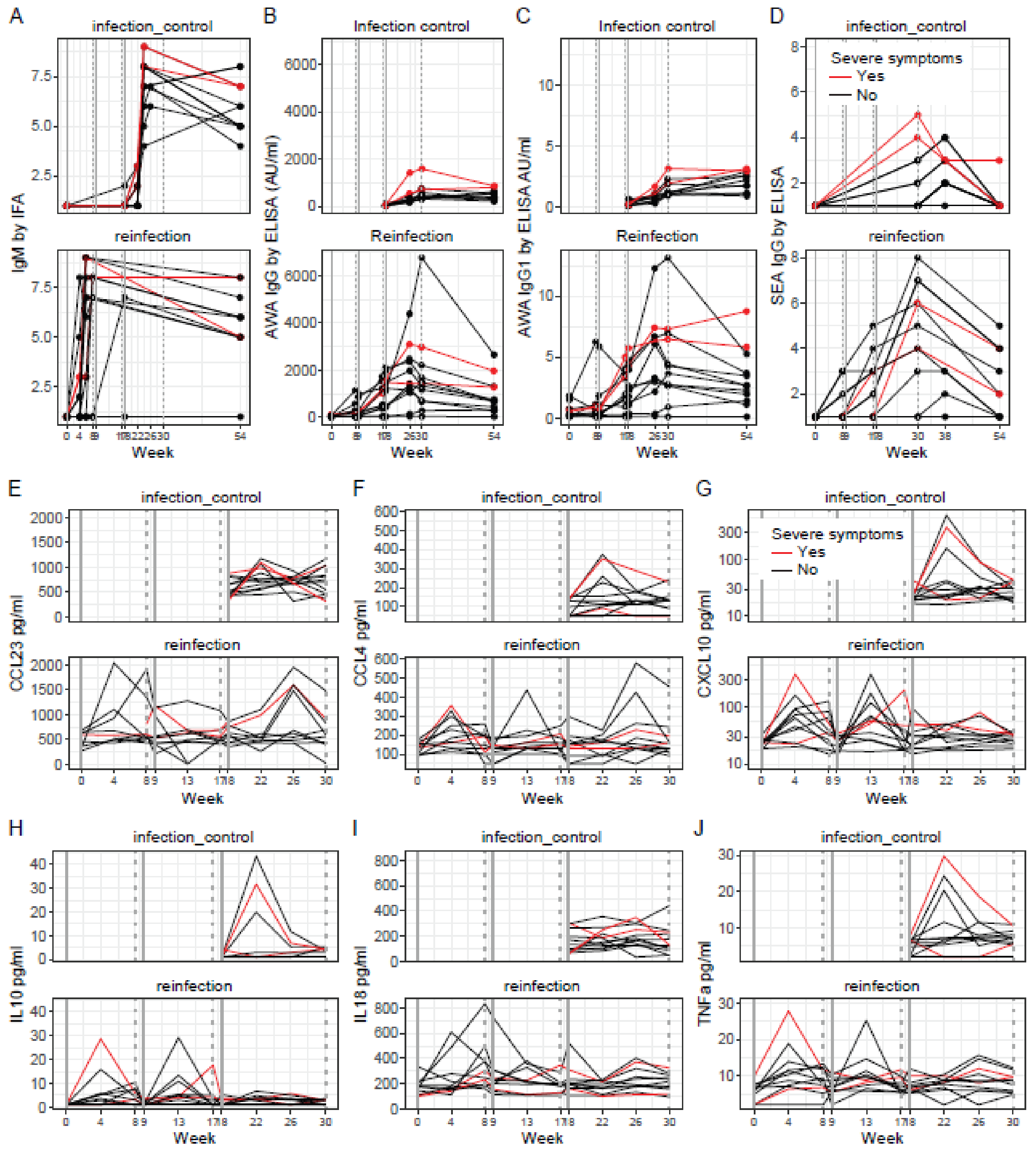


Plots show the individual changes in antibody levels in worm-specific IgM (A), AWA-specific IgG (B), AWA-specific IgG1 (C), and SEA IgG (D). For CCL23 (E), CCL4 (F), CXCL10 (G), IL-10 (H), IL18 (I), and TNF (J) individual participant data are plotted. Data is stratified for severe acute schistosomiasis symptoms (red, yes, n=4). The solid, grey vertical line shows Sm exposure weeks (0,9,18), while the dotted, grey vertical line shows when PZQ treatment was given (8,17,30). AWA = adult worm antigen; SEA = soluble egg antigen
